# Financial Feasibility of Developing Sustained-Release Incrementally Modified Drugs in Thailand’s Pharmaceutical Industry: Mixed Methods Study

**DOI:** 10.2196/65978

**Published:** 2025-07-01

**Authors:** Manthana Laichapis, Rungpetch Sakulbumrungsil, Khunjira Udomaksorn, Nusaraporn Kessomboon, Osot Nerapusee, Charkkrit Hongthong, Sitanun Poonpolsub

**Affiliations:** 1 Department of Social and Administrative Pharmacy, Faculty of Pharmaceutical Sciences, Chulalongkorn University, 254 Phayathai Road, Pathum Wan, Bangkok, 10500, Thailand, 66 8-860-318-88; 2 Department of Social and Administrative Pharmacy, Faculty of Pharmaceutical Sciences, Prince of Songkla University, Songkla, Thailand; 3 Department of Social and Administrative Pharmacy, Faculty of Pharmaceutical Sciences, Khon Kaen University, Khon Kaen, Thailand; 4 Food and Drug Administration Thailand, Nonthaburi, Thailand

**Keywords:** financial, economics, R&D, research and development, surveys, interviews, costs, revenue, policies, drugs, pharmaceuticals

## Abstract

**Background:**

Thailand’s pharmaceutical industry is prioritizing innovation and self-reliance through the development of incrementally modified drugs (IMDs), particularly sustained-release dosage forms. However, the financial feasibility of IMD development remains underexplored.

**Objective:**

This study evaluates the financial feasibility of developing sustained-release IMDs in Thailand, focusing on costs, timelines, and investment requirements to inform strategic decision-making.

**Methods:**

A mixed methods approach was used, combining literature reviews, expert interviews, and financial modeling. Two scenarios were analyzed: (1) only development (phase I) and (2) full clinical trials (phase I to III). Sensitivity analysis was used to assess the impact of key variables on financial feasibility.

**Results:**

The research and development (R&D) process for sustained-release IMDs takes 7 years for phase I–only development, costing US $1.46‐3.09 million, and 11 years for full clinical trials, costing US $18.60‐20.23 million. Process validation batches accounted for 60% of costs in phase I–only scenarios, while clinical trials represented 70% of costs in full clinical trial scenarios. The annual income required for a 5-year payback period ranged from US $0.20‐1.80 million (phase I only) to US $3.01‐27.11 million (full trials). Shorter R&D durations and longer payback periods substantially improved feasibility.

**Conclusions:**

Developing sustained-release IMDs in Thailand involves substantial costs and extended timelines but offers a lower-risk alternative to new chemical entities. Strategic investments, efficient R&D processes, and supportive policies are essential to enhance feasibility and alignment with national goals of innovation and self-reliance.

## Introduction

The Thai pharmaceutical industry is undergoing significant transformation in alignment with Thailand’s Pharmaceutical Development Action Plan (2023‐2027), which builds upon the foundation of earlier policies, including the National Strategic Master Plan (2018‐2037). These initiatives collectively emphasize enhancing the national drug system, fostering domestic pharmaceutical manufacturing capabilities, and achieving self-reliance through sustainable development [1-3]. The current action plan (2023‐2027) prioritizes accelerating the industry’s capabilities in research, development, and production of vaccines, drugs, herbal products, and biologics, while promoting local pharmaceutical industries to reduce import dependency and increase export potential. By focusing on innovation, technological advancement, and strategic investments, this plan ensures the industry’s alignment with national health care priorities and global market demands, driving growth and competitiveness in the pharmaceutical sector.

Currently, Thailand’s pharmaceutical manufacturing industry is predominantly focused on the production of generic drugs, with an average of 540 generic drug approvals annually, including approximately 35 new ones [3]. However, the development of new chemical entities (NCEs) remains limited due to challenges such as insufficient investment, lack of advanced technology, and a shortage of specialized talent. Given these constraints, a more feasible approach for Thailand’s pharmaceutical sector lies in the development of incrementally modified drugs (IMDs). IMDs involve enhancing existing drugs through modifications in delivery systems, indications, combinations, administration routes, dosage forms, and strengths, offering a pathway to sustainable self-reliance while reducing costs and risks associated with NCE development [4].

Globally, IMDs have gained traction in high-income countries, with high listing and reimbursement rates, demonstrating their potential to improve patient outcomes and health care efficiency [5]. In Thailand, focusing on IMDs aligns with the country’s strategic goals of fostering innovation, reducing reliance on imported pharmaceuticals, and enhancing the competitiveness among local manufacturers. By leveraging advanced technology platforms, the development of IMDs can provide a viable pathway for the Thai pharmaceutical industry to achieve greater self-sufficiency and contribute to the broader health care system.

This study aims to examine the financial feasibility of developing IMD dosage forms within Thailand’s pharmaceutical manufacturing industry. By evaluating the economic viability and potential return on investment of IMDs, this study will provide evidence-based insights to support decision-making and guide strategic investments in the sector. The findings may provide a foundation for policy makers and stakeholders in formulating targeted strategies to promote innovation in IMDs, enhance domestic pharmaceutical capabilities, and reduce reliance on imported drugs. Ultimately, the study may also contribute to strengthening Thailand’s pharmaceutical sector, ensuring its alignment with national development goals and global health care trends.

## Methods

### Study Design

This study used a mixed methods approach, combining qualitative and quantitative components. Given the lack of publicly available data on IMD development, this approach was necessary to triangulate data from multiple sources, ensuring robustness and reliability. The qualitative component included a literature review, surveys, and expert interviews, while the quantitative component focused on financial modeling and analysis.

### Data Collection

#### Literature Review

A comprehensive review of existing IMD dosage forms, manufacturing processes, cost structures, regulatory requirements, and market trends was conducted using PubMed, Scopus, and industry reports. This review served as input for the development of the financial model and interview guide.

#### Survey

A survey was designed to estimate costs associated with IMD development. The cost structures were adapted from a prior study on the impact of the Thai–European Union (EU) free trade agreement (FTA) on the pharmaceutical supply chain in Thailand [6]. Cost collection forms were sent to five IMD experts for feedback, refinement, and validation, after which cost estimates were provided.

#### Interviews

Snowball sampling was used to identify participants due to the specialized nature of IMD development and the limited number of manufacturers in this field. This approach allowed the research team to access experts with relevant knowledge and experience in IMD development.

Semistructured interviews were conducted with 15 experts, including company owners, industry leaders, policy makers, and researchers. Interviews continued until data saturation was achieved, with no new themes emerging.

Interviews were conducted online and recorded with participants’ consent. The key interview questions were focused on costs associated with research and development (R&D), manufacturing technology, and clinical and nonclinical studies. Data saturation was achieved when no new themes emerged. To ensure transparency and reproducibility, detailed descriptions of the interview guide and survey questions are provided in Multimedia Appendix 1.

### Ethical Considerations

The study received ethical approval from the Research Ethics Review Committee for Research Involving Human Subjects, Health Science Group at Chulalongkorn University, Thailand (COA No. 176/2564). We confirm that participation in the online Zoom sessions was entirely voluntary. Participants were informed in advance about the purpose and format of the sessions, and they had the right to decline participation without any consequences. Choosing to join the Zoom session was considered as implied consent by action, in alignment with ethical practices for minimal-risk research. To protect participant confidentiality, no personally identifiable information was recorded during the sessions. All data were de-identified prior to analysis. Participants received approximately $30 USD as compensation for their time and contribution, ensuring transparency and fairness throughout the research process.

We confirm that participation in the online Zoom sessions was entirely voluntary. Participants were informed in advance about the purpose and format of the sessions, and they had the right to decline participation without any consequences. Choosing to join the Zoom session was considered as **implied consent by action**, in alignment with ethical practices for minimal-risk research.

### Data Analysis

#### Financial Model Development

Two financial model scenarios were developed to assess IMD feasibility, focusing on IMD types and cost estimation. Sustained-release formulations, which are the most preferred type of IMDs by the domestic pharmaceutical industry, were selected based on results from a prior feasibility study [7]. The cost estimation was adapted from the Thai-EU FTA study [6], covering sourcing, R&D (ie, laboratory scale, pilot batch, and stability studies), nonclinical and clinical trials, and registration and process validation [8,9]. Costs were estimated under two regulatory scenarios: (1) conducting only phase I clinical trials and (2) conducting full clinical trials [10].

#### Sensitivity Analysis

A sensitivity analysis was conducted to evaluate the impact of variations in key variables including cost, duration, and payback period on the financial feasibility of IMD development. This analysis provided insights into the robustness of the financial models under varying assumptions.

## Results

### Overview

A prediction market analysis by Hongthong et al [11] on the feasibility of IMD development by the domestic pharmaceutical industry identified sustained-release dosage forms as the most preferred option, which guided the financial feasibility analysis in this study [7,12]. The assumptions and input data used for this analysis are detailed in Table 1.

**Table 1.** Input data and assumptions for the financial model and financial feasibility study.

Variables	Assumptions	Source of data
Cost of sales	25% of revenue	Jiang et al [13]
Operational expense	40% of revenue	Jiang et al [13] and interviews
Discounted rate	Discount rate of 3%	Haacker et al [12]
Interest rate	Interest rate for business is 3%	Interviews
Tax rate	Corporate tax rate is 20%	IDRG Consultancy Services [14] and interviews
Expected payback period	Payback period that investors could accept is 5‐10 years	Interviews

### Financial Feasibility Analysis Model

To assess the financial viability of investing in the development of sustained-release dosage forms, a financial feasibility analysis model was developed. This model calculates the payback period and market growth rate based on two primary components (ie, cost and revenue components). The cost component estimates the expenses associated with R&D of the new dosage form, while the revenue component forecasts the income required to achieve a return on investment within a specified payback period. The model’s flexibility allows for adjustments in key variables such as the payback period and market growth rate, enabling stakeholders to make informed strategic decisions regarding pharmaceutical R&D investments.

### Cost Analysis for Sustained-Release Dosage Form Development

Table 2 presents the cost analysis for two development scenarios. In scenario 1, which involves only phase I clinical studies, the R&D process for new sustained-release IMD formulations was estimated to take 7 years, with development costs ranging from US $1.46 to 3.09 million. Approximately 60% of the total costs were allocated to process validation batches, a critical step requiring three consecutive production batches. This phase represents a significant capital investment, with varying costs depending on production complexity. In scenario 2, which includes full clinical trials from phase I to phase III, the development duration extended to 11 years, with fixed costs ranging from US $18.60 to 20.23 million. In this case, 70% of the total R&D budget was dedicated to clinical studies, which are essential for demonstrating the efficacy and safety of new drugs.

The sensitivity analysis presented in Table 3 evaluates the financial implications under different scenarios. For scenario 1, which involves only phase I studies with R&D costs of US $1.46 million, the annual income required to recover the invested capital—assuming a 5-year payback period—ranges from US $0.20 to 1.80 million. In contrast, for scenario 2, which includes full clinical trials, the required annual income increases substantially, ranging from US $3.01 to 27.11 million.

**Table 2.** The process and cost of developing IMDs^a^ in a sustained-release form by the domestic pharmaceutical industry.

Process	Details [6]	Scenario 1 (US $, in millions)	Scenario 2 (US $, in millions)	Information source
Sourcing	Local manufacturers choose reference IMDs based on marketing, user needs, sales, patents, and suitability.	0.02	0.02	This study
R&D^b^ laboratory scale	The R&D department conducts laboratory-scale studies to develop suitable formulations for sustained-release drugs, including analytical method development and determination of finished product specifications (FPS).	0.06‐0.15	0.06‐0.15	This study
Pilot scale	After successful drug R&D, pilot batch production begins, followed by stability studies to determine shelf-life specifications. Results are reported to the FDA^c^.	0.27‐0.66	0.27‐0.66	Sertkaya et al [15] and this study
Clinical study				
Phase I	Samples from the pilot batch production will be sent to study the effect of food on bioefficacy through a bioequivalence study and evaluating the effect of alcohol on dose dumping.	0.29	0.29	Thai FDA [10], National Institute of Health [16], DiMasi et al [17], and this study
Phase II	In case the pharmacokinetics of a new drug are clinically significantly different from the reference drug, phase II and III studies of the new drug may be necessary.	—^e^	4.29	Thai FDA [10] and this study
Phase III	In case the pharmacokinetics of a new drug are clinically significantly different from the reference drug, phase II and III studies of the new drug may be necessary.	—	12.86	Thai FDA [10] and this study
Registration	Registration of new drug formulas follows the ASEAN^d^ harmonization criteria. Application documents included administration data, product information, quality, safety, and efficacy parts. The nonclinical and clinical study data can refer to recommendations and guidelines.	0.003	0.003	This study
Process validation batch	After obtaining FDA registration, drugs can be produced in commercial batches. Process validation and inspection results are submitted for permission to continue production and distribution.	0.81‐1.97	0.81‐1.97	This study
Total cost	—	1.46‐3.09	18.60‐20.23	—

a IMD: incrementally modified drug.

b R&D: research and development.

c FDA: Food and Drug Administration.

d ASEAN: Association of Southeast Asian Nations.

e Not applicable.

**Table 3.** Expected annual revenue after product launch of both scenarios (US $ [in millions]/year).

Expected payback	Annual revenue (US $ [in millions]/year)									
	Year 1	Year 2	Year 3	Year 4	Year 5	Year 6	Year 7	Year 8	Year 9	Year 10
Scenario 1^a^										
5-year period	0.20	0.40	0.80	1.20	1.80	—^c^	—	—	—	—
10-year period	0.04	0.08	0.17	0.25	0.38	0.49	0.61	0.77	0.96	1.20
Scenario 2^b^										
5-year period	3.01	6.03	12.05	18.08	27.11	—	—	—	—	—
10-year period	0.63	1.25	2.50	3.75	5.63	7.32	9.15	11.44	14.30	17.87

a The parameters of the base case analysis for scenario 1 are that the duration of research and development is 7 years, and the research and development cost is US $1.46 million.

b The parameters of the base case analysis for scenario 2 are that the duration of research and development is 11 years, and the research and development cost is US $18.60 million.

c Not applicable.

The duration of the R&D process substantially impacts financial expenditures; shorter R&D periods can reduce costs and enhance project feasibility. High-risk activities such as complex formulation and analytical method development often involve a higher likelihood of failure and require advanced clinical studies, necessitating larger capital investments. Therefore, well-planned R&D processes can substantially reduce costs and improve investment returns.

The analysis also examines the impact of extending the payback period to 10 years, which lowers the capitalization point and reduces the annual income required. This consideration is particularly relevant for drugs targeting chronic diseases, which typically have longer market life cycles. Additionally, factors such as annual sales growth rates upon launch, the competitive landscape, and government regulations critically influence the financial feasibility of developing sustained-release IMDs [15].

## Discussion

### Principal Findings

This study systematically explores the financial viability and strategic implications of developing IMDs, with a focus on sustained-release dosage forms. Our analysis highlights the financial and investment requirements for launching IMDs into the market, particularly in comparison to generic drugs.

Developing IMDs, particularly as sustained-release formulations, is substantially more resource-intensive and time-consuming than producing generic drugs. The extended timelines and higher costs are primarily attributed to the complexities of modifying and validating existing drugs, which necessitate extensive clinical testing. In contrast, Liangrokapart et al [6], in a study on the impact of the Thai-EU FTA concerning intellectual property rights on the pharmaceutical supply chain in Thailand, suggested that generic drug development typically required 25 to 46 months, with considerably lower R&D costs ranging from US $0.19 to 1.13 million [6].

A key challenge in this analysis is the absence of specific active ingredient data, which complicates accurate forecasting of market growth and sales revenue. Despite these limitations, the chosen methodology effectively captures the financial intricacies of IMD development, providing robust insights into the associated costs and investment requirements.

Compared to the development costs of NCEs reported in previous studies—including an analysis by Sertkaya et al [15] on drug development costs in the United States (2000‐2018) and the study by DiMasi et al [18] on the price of innovation and research on R&D costs and returns by therapeutic category—the costs for IMDs are substantially lower. This is primarily because IMDs do not incur discovery and preclinical expenses. Additionally, IMDs have lower failure rates than NCEs, suggesting a potentially lower-risk investment profile. This aligns with findings from a study on IMDs under the USFDA 505(b)(2) NDA pathway, which reported clinical trial completion times of 12‐24 months and development costs of US $2 to 10 million, closely mirroring the outcomes of this study [4].

The findings from both scenarios underscore that IMD development entails higher costs and longer timelines compared to generic drugs. These challenges stem from the need to develop new formulations and conduct comprehensive clinical studies. However, shorter R&D periods can substantially reduce costs and enhance project feasibility, emphasizing the importance of efficient R&D planning.

The early stage and inexperience of IMD development within the domestic industry may result in longer timelines and elevated costs. Limited domestic expertise, coupled with the complexities of clinical trials and regulatory processes, poses additional challenges. The high investment required for IMD development necessitates a strong focus on market feasibility and sales potential, particularly in a competitive landscape dominated by generic drugs.

While this study offers valuable insights into the financial feasibility of IMD development in Thailand, several limitations must be acknowledged. First, cost estimates were derived from expert feedback and prior studies, which may not fully capture the variability inherent in real-world manufacturing processes. Finally, the findings may be context specific and not directly applicable to other types of IMDs or pharmaceutical markets.

For the future direction of this research, IMDs represent an incremental innovation that can be developed in various forms, including stand-alone and combination products. Therefore, further studies are needed to assess the feasibility of developing different types of IMDs to enhance patient health outcomes and quality of life.

Additionally, the regulatory process and guidelines play a crucial role in IMD development, making it necessary to study the impact of regulatory changes on IMDs. Furthermore, the pricing and reimbursement mechanisms for IMDs remain unclear for the local pharmaceutical industry, highlighting the need for further exploration of this topic.

### Conclusions

This study provides essential insights into the financial aspects of developing sustained-release IMDs in Thailand, highlighting the extensive resources and strategic planning required. These findings underscore the complexity of predicting financial outcomes due to the variability in active ingredients and market dynamics. Although the development of IMDs involves substantial investment and extended timelines, understanding these financial and operational dimensions is crucial for successful drug development. Future research should further investigate the full cost spectrum of various types of IMD approaches to enhance the financial predictability and success of these studies.

## Supplementary material

10.2196/65978 Multimedia Appendix 1 Survey instrument and interview guide for the financial feasibility study of sustained-release incrementally modified drugs in Thailand.

## Abbreviations

EU: European Union
FTA: free trade agreement
IMD: incrementally modified drug
NCE: new chemical entity
R&D: research and development
