# Supplementary material for: Financial Feasibility of Developing Sustained-Release Incrementally Modified Drugs in Thailand’s Pharmaceutical Industry: Mixed Methods Study
Source: JMIRx Med. 2025 Jul 1;6:e65978. doi: 10.2196/65978 (PMC12236265; doi:10.2196/65978)
Supplement: Multimedia Appendix 1 [file xmed-v6-e65978-s001.docx]

### Survey

The survey method was used to identify and estimate costs based on the defined cost structures, which were derived from the 'Impact of Thai-EU Free Trade Agreement (FTA) concerning Intellectual Property Rights on the Pharmaceutical Supply Chain in Thailand' study [1]

1. The collection forms with pre-defined cost structures were sent to 5 IMDs (Industrial Manufacturing and Development) experts.
2. The experts provided estimated costs and comments to make the cost structure more valid.

### Interview

The interview process involved selecting experts and individuals with specialized knowledge in assessing the IMDs manufacturing landscape in Thailand. Participant recruitment took place from August 5, 2021, to August 4, 2022, marking the period during which individuals were actively enrolled in the study.

A.) Interview process

1. The researcher sent interview questions to research participants and schedule individual online interviews. Each interview is expected to take approximately 1 hour. The researcher sought permission from participants to record the interview for data analysis purposes (audio recordings will be destroyed at the end of the project).
2. Participants' agreement to participate in the research and their decision to participate in the online interview were considered as consent. Participants were not required to sign a letter of consent.
3. Participants who complete an online interview received remuneration of 1,000 baht per person.

B.) Question guidelines:

1. What are the costs associated with the research and development of a dosage form for IMDs?
2. What are the procedures involved in the research and development of a dosage form for IMDs, and what are the associated costs?
3. What are the costs related to manufacturing technology?
4. What are the costs of conducting clinical and non-clinical studies?

1. Liangrokapart J, Kessomboon N, Sakulbumrungsil R, Akaleephan C, Poonpolsub S, Saerekul P. Impact of Thai-EU Free Trade Agreement (FTA) concerning Intellectual Property Rights on the Pharmaceutical Supply Chain in Thailand. 2013.
